# Chemoprevention of Head and Neck Cancers: Does It Have Only One Face?

**DOI:** 10.1155/2018/9051854

**Published:** 2018-09-25

**Authors:** Krzysztof Siemianowicz, Wirginia Likus, Mariola Dorecka, Renata Wilk, Włodzimierz Dziubdziela, Jarosław Markowski

**Affiliations:** ^1^Department of Biochemistry, School of Medicine in Katowice, Medical University of Silesia, Medyków 18 Str., 40-752 Katowice, Poland; ^2^Department of Anatomy, School of Health Sciences in Katowice, Medical University of Silesia, Medyków 18 Str., 40-752 Katowice, Poland; ^3^Department of Ophthalmology, School of Medicine in Katowice, Medical University of Silesia, Ceglana 35 Str., 40-952 Katowice, Poland; ^4^Outpatient Clinic for Treatment of Chronic Pain, Wyszyńskiego 12 Str., 41-200 Sosnowiec, Poland; ^5^Department of Laryngology, School of Medicine in Katowice, Medical University of Silesia, Francuska 20/24 Str., 40-027 Katowice, Poland

## Abstract

Head and neck squamous cell cancer (HNSCC) represents a significant burden worldwide. Chemoprevention of HNSCC is a means of cancer control with a use of drugs or natural agents in order to hinder or delay the cancer development. The purpose of this article is to review mechanism of action of different chemopreventive agents' groups and results of most important researches concerning them. The safety issues of HNSCC chemoprevention are also discussed. In case of HNSCC there is currently no agent, which would give positive result in the third phase of clinical trials. Promising results of preclinical trials are not always confirmed by further tests. Main problems are low effectiveness, high toxicity, and lack of highly specificity biomarkers for monitoring the research. New trials concerning many agents, as well as novel technologies for provision of pharmaceutical forms of them, including drug nanocarriers, are currently underway, which gives hope for finding the perfect chemopreventive agent formula.

## 1. Introduction

The name “head and neck cancers” comprises carcinomas located in the head and neck area, excluding brain tumours. Those carcinomas have various locations: larynx, mouth cavity, nasal cavity, lips, nasal/paranasal sinuses, tongue, and salivary glands. In 2018, the number of new cases of oral cavity and pharynx cancers in the USA is assessed to amount to 51,540 and larynx cancers to 13,150, while the number of deaths caused by them is estimated to be 10,030 and 2,970, respectively. On the other hand, in 2016 the number of male oral cavity and pharynx cancer survivors was estimated to reach 229,880, and it is predicted that by the year 2026 it will have reached 293,290. The estimations for the year 2020 assume as many as 833,000 new incidents worldwide, of which 151,000 will be in Europe, amounting to 5% and 4%, respectively, of all new incidents of cancer. A vast majority, amounting to over 90% of all cases, are head and neck squamous cell carcinomas (HNSCC). The five-year survival rate among patients with head and neck squamous cell carcinoma amounts to some 40-50% [1–4].

The risk factors which conduce the development of such diseases are exposure of mucous membrane to tobacco smoke and spirits, in particular the synergistic action of those factors, viral infections with HPV or EBV, exposure to harmful environmental factors at work, previous radiotherapy, insufficient hygiene of the mouth cavity, prolonged mechanical irritation by improperly made dentures, and immunosuppression, as well as improper diet leading to vitamin deficiency [5–8]. The anatomical region which is the focus of this paper is the crossroads of alimentary and respiratory tracts. Because squamous cell carcinoma occurring in the head and neck area may involve structures of respiratory as well as alimentary tract, oftentimes the neoplastic lesions in that anatomic location are referred to as “upper aerodigestive malignancies” [9].

HNSCC in some 20% of the cases originate from precancerous lesions. Those precancerous lesions, according to WHO definition, are morphological lesions, related to increased risk of malignant neoplasm to occur. Premalignacies for HNSCC comprise mainly leukoplakia, sometimes referred to as “smoker's keratosis” and erythroplakia, as well as chronic inflammation and hypertrophic papillomas of adult type. Leukoplakia is manifested, macroscopically, as elevated white patches. The microscopic image shows leukoplakia as demonstrating hyperplasia, hyperkeratosis, or parakeratosis. Those lesions may be accompanied by dysplasia of various degrees. Erythroplakia macroscopically presents as diffused, multifocal superficial lesions, or slightly depressed lesions having red or velvety-red colour. Microscopic images show pleomorphic squamous cells with cellular atypia comprising the entire thickness of the mucosa. It can be considered to be carcinoma* in situ* [9, 10]. According to some authors, erosive lichen planus should be considered the third kind of premalignancy. All the lesions mentioned demonstrate heterogeneity regarding anatomic location, colour, and texture [11].

From the perspective of histopathology, dysplasia is a premalignancy. Dysplasia is the occurrence of irregular/immature cells in the epithelium, cells that have changed morphology (polymorphic cells with big irregular nuclei, containing an increased number of nucleoli), and aberrations in the laminar structure of the epithelium (chaotic, irregular arrangement of cells, change in the polarity of basilar cells, and the presence of incorrect mitoses above the basal layer). A part of those lesions may be reversible and may subside spontaneously. The presence of premalignant condition does not imply that a neoplasm will develop for sure; it only results in increased risk of its formation [12]. For that reason, some authors refer to them as oral potentially malignant lesions (OPMLs). The estimations concerning the percentage of those lesions which turn malignant and the percentage of HNSCC preceded with premalignancies vary much and, according to various authors, may range from 1.1% to 25% [11, 13–15]. OPMLs have different potential for neoplastic transformations, depending on other factors: clinical, demographic, etiologic, histological, and molecular ones [16]. The basis for morphological changes is the genetic changes of cells. Gradual accumulation of gene mutations, the products of which participate in controlling the processes of growth, differentiation, and apoptosis, as well as DNA replication and repair, is the cause of changes taking place in cell phenotype.

## 2. Chemoprevention

Hopes for arresting the development of neoplastic disease arise from research concerning chemoprevention. Chemoprevention implies the application of natural or pharmacological factors, in order to slow down, restrain, or cause regression of the cancerogenic process in people with elevated risk of neoplastic disease. The authors of the concept of neoplasm chemoprevention are L.W. Wattenberg and M.B. Sporn. Their studies made in the 1960s and 1970s provided the basis for further research in that respect [17, 18]. Extending the knowledge concerning the causes of cancerogenesis on molecular level created the basis for trying to find specific chemopreventic agents. Due to the mechanism and stage, at which chemopreventive agents act, they may be divided into blocking (anti-initiative) and suppressive ones (Table 1). Because some compounds may act both at the stage of initiation and promotion of carcinogenesis, the division that is more often applied is not based on the stage of tumour development, but on the basis of the cascade of events which they interfere with [19].

One can distinguish three main strategies of chemoprevention: the primary one (anti-initiative), secondary one, and tertiary one. Primary chemoprevention is addressed to healthy individuals with elevated risk of cancer development, with reference to the population of, e.g., those exposed to carcinogen action, at present or in the past. Examples of primary chemoprevention include HPV vaccination and discontinuation of tobacco smoking. Secondary chemoprevention has the task of inhibiting the progress of existing premalignancies. Tertiary chemoprevention concerns prevention of second primary tumours (SPTs) as well as recurrence in patients who have gone successfully through the treatment of squamous cell carcinoma lesion [19].

Some chemical compounds have been tried for application in both chemoprevention and in the treatment of carcinomas. Depending on the fact whether a given chemical substance is used in treatment of cancer or in its prevention and the type of prevention, the balance of gains and losses resulting from a given intervention change and—what follows—the acceptable adverse effects and permissible toxicity level also vary, as well as the permissible maximum dose resulting from that.

Besides the notion of cancer chemoprevention, one can also encounter the term biochemoprevention. It implies combining, in prevention, a chemical substance with a biological factor, most often with interferon-*α* [20].

### 2.1. Retinoids

Retinoids constitute a group of chemical compounds comprising vitamin A, the natural analogs of vitamin A, and its synthetic derivatives (Table 2). Oftentimes they are mistaken for carotenoids, as carotenoids have a similar chemical structure. Of the about 600 compounds which may be classified as carotenoids, some 50 may be metabolized to vitamin A, being its provitamins. For humans, the most valid carotenoid is *β*-carotene [9].

The first observations concerning the role of retinoids in chemoprevention go back to 1925, when Wolbach and Howe noticed that, in rodents with vitamin A deficiency, a reversible squamous metaplasia of the lung occurs after its administration, similar to the metaplastic lesions diagnosed in smokers. Thirty years later, in 1955, Lasnitzki demonstrated that experimentally induced premalignancies subside under hypervitaminosis A [9, 21]. In 1979 Merriman confirmed the importance of therapy with retinoids in inhibition of carcinogenesis and—what is more—demonstrated that discontinuation of such treatment causes further progress of neoplasia [9, 22].

Retinoids bind with specific nuclear receptors: RAR (retinoid acid receptor) and RXR (retinoid X receptor), which perform the function of transcriptive factors. After binding the retinoid with the receptor, the RARE (retinoid acid response element) sequences may be launched, which are present in promoter areas, as well as RXRE (retinoid X response element), which are adjacent to some 100 genes and may influence their expression. This mechanism controls the development and differentiation of both regular and neoplastic cells. Retinoids inhibit the expression of transcriptive factor AP-1 (activator protein 1), enhance the expression of TGF-*β*2 (transforming growth factor *β*2), modulate histone acetylation, and may induce apoptosis [23, 24].

RAR receptors bind with all the trans-retinoic acids, whereas RXR receptors bind only with the 9-cis-retinoic acid. Both receptors occur in three different forms: *α*, *β*, and *γ*. The RXR receptor forms a RXR-RXR homodimer or RXR-RAR heterodimer, unlike the RAR receptor, which forms only a heterodimer. Both dimers demonstrate a significant affinity to the sites of responses within DNA of the genes regulated by retinoids. Synthetic retinoids usually demonstrate selectivity towards the type of receptor, RAR or RXR, or even their definite forms [25].

The first studies concerning chemoprevention focused upon attempts of reversing the premalignancy lesions in the mouth cavity (mainly leukoplakia and submucous fibrosis) as those lesions may easily be subject to biopsy and observation of the clinical condition. The efficiency of treating leukoplakia with 13-cis-retinoic acid (isotretinoin) has been demonstrated and proven by Hong [26]. The compound was administered in the doses of 1-2 mg/kg of body mass/day, for 3 months, which resulted in reduction of the size of leukoplakia foci in 67% of subjects and regression of dysplasia in 54% of patients. Sankaranarayanan et al. [27] examined 160 subjects, who have been divided into 3 groups. For 12 months, the first group was administered vitamin A in the doses of 300 000 IU/ week, the second received *β*-carotene in the doses of 360 mg/week, and the third was administered placebo. Promising results were obtained, particularly in the group receiving vitamin A (lesions subsided in 52% of subjects from group one, 33% of subjects from group two, and 10% from the group receiving placebo). Those promising results provided the basis for studies concerning the application of retinoids in tertiary prevention that is prevention of SPTs occurrence and recurrence of the disease in patients who successfully completed squamous cell carcinoma treatment.

The study took place in 1990 [28] and comprised a group of patients with head and neck squamous cell carcinomas, previously treated successfully by means of surgery, radiotherapy, or both those methods. It comprised 103 patients, who were divided into 2 groups and received 13-cis-retinoid acid in the doses of 50 to 100 mg per 1 m^2^ of body surface/day or placebo, for one year. After 3.5 years, no differences were observed as to the number of recurrences of primary cancer; still a lower amount of SPTs was noted in the group receiving isotretinoin (4%), in comparison with the group receiving placebo (24%).

The studies conducted by Papadimitrakopoulou et al. indicated higher efficiency of isotretinoin, administered for 3 months, in lower doses of 0.5 mg and 0.25 mg/kg of body mass/day, as compared with vitamin A and vitamin A combined with beta-carotene, in the treatment of oral cavity leukoplakia. Oral cancer-free survival was similar in all three groups of patients. Adverse effects were significantly more frequent in the group receiving isotretinoin [29]. A randomized study conducted by Khuri et al. in 2005 also failed to produce expected results. That study comprised 1190 subjects, who underwent successful treatment of head and neck squamous cell carcinoma, stage I or II. Those patients received isotretinoin, in the doses of 30 mg/day or placebo, for three years. No statistically significant differences were demonstrated, either in the number of SPTs or in survival [30].

In a phase 3 study concerning the application of low doses of isotretinoin (7.5 or 10 mg, depending on body weight) to 176 patients after treatment of HNSCC, stage I or II, and comparing with placebo, no statistically significant differences were found, as regards the occurrence of SPTs, or prolongation of survival [31]. A large EUROSCAN study (European Study on Chemoprevention with Vitamin A and N-acetylcysteine) comprising 2592 individuals was aimed to check if vitamin A and/or N-acetylcysteine administered for two years could protect against cancers induced by tobacco smoking (i.e., HNSCC and lung cancer). After a median follow-up of 49 months there was no difference in overall survival or event-free survival between individuals receiving each agent separately, receiving both agents, and control group receiving no chemoprevention [32].

In the study conducted by Kadakia et al., comprising 143 patients treated for head and neck carcinoma, isotretinoin was applied in the form of mouth-wash. The mouth-wash was applied to patients for 12 months, while the follow-up period was 24 months. Over that time, all forms of cancer relapse were noted, as well as occurring neoplastic lesions* in situ* and dysplasias. Mouth-wash with isotretinoin turned out to be an efficient form of chemoprevention in patients with multifocal lesions in the mouth cavity, of low level of disease advancement, who had previously undergone surgical resection of early cancer, and with multifocal dysplasia of medium or low level, treated with CO_2_ laser ablation. On the other hand, it turned out to be ineffective in patients with dysplastic lesions, after previous cancer resection surgeries, and in patients after* in situ* resection of neoplasm [33].

### 2.2. COX-2 Inhibitors

In head and neck carcinomas, increased production of prostaglandins has been detected, particularly of prostaglandin E_2_ (PGE_2_), in comparison with healthy tissue. Cyclooxygenase (COX) catalyzes the first stage of transformation of arachidonic acid, common for the pathway of prostaglandin, prostacyclin, and thromboxane synthesis. In the second pathway of arachidonic acid metabolism, initiated by lipoxygenases, leukotrienes are formed. Cyclooxygenase is present in two main isoenzymes, COX-1 and COX-2. COX-1 is an enzyme which is constitutively synthesized, and is responsible for the synthesis of those prostaglandins, which control certain physiological processes, e.g., gastric juice secretion, or aggregation of platelets, as well as for the synthesis of prostacyclin and thromboxane. COX-2, on the other hand, is an enzyme whose synthesis is induced by proinflammatory cytokines and growth factors, which increase its synthesis in tissues in case of inflammatory condition or neoplasm. In most healthy tissues this enzyme is not detected. Increased activity of this enzyme has been confirmed, among other things, in head and neck squamous cell carcinomas, as well as in dysplastic lesions in that area. Prostaglandins synthesized by COX-2 stimulate the proliferation of cells, act as immunosuppressants (which minimize the chances of the immune system to destroy irregular cells), as well as promote angiogenesis and metastasis. Increased synthesis of PGE_2_, referred to above, results in reduced frequency of apoptosis [34]. In connection with that, COX inhibitors started to be studied as chemopreventive factors [35]. Nonsteroid anti-inflammatory drugs (NSAIDs) inhibit both isoenzymes, which is demonstrated by adverse effect, among others, on the alimentary tract mucosa, thus promoting the development of gastric ulcers and in some case causing bleeding from the ulcers. For that reason, selective inhibitors of COX-2 have been introduced to the market, as anti-inflammatory drugs deprived of that adverse effect. Celecoxib was one of the first such compounds. The research concerning its application as a chemopreventive agent in head and neck squamous cell carcinomas resulted in contradictory conclusions, what is more, the studies were conducted on relatively small groups. In the study conducted on 22 patients with premalignancies in the mouth cavity, the level of PGE_2_ was monitored, as well as its changes in response to the administered celecoxib. Biopsies of the morbid tissues were performed: the first one after 12 weeks of treatment,and another one after 12 months of drug administration. Reduced level of PGE_2_ was noted, as well as less dysplasia [36]. In the randomized phase 2 study, with 50 subjects, the first group of patients received celecoxib in the doses of 100 mg, twice a day; in the second group the drug dose was 200 mg, also administered twice daily; the third group received placebo. Like in the previous study, the subjects had confirmed premalignancies of the mucous membrane in the oral cavity. Unfortunately, after 12 weeks of the study, no advantageous, chemopreventive action was observed in study subjects [37]. In order to eliminate the possible adverse effects, also a topical application of the drug was tested, which may be applied for a longer time, without risk for the cardiovascular system. In the randomized study, flushing of the mouth cavity with selective inhibitor of COX-2 (ketorolac) was applied. The number of study subjects was 57; they flushed the oral cavity with 10 ml of 0.1% solution of ketorolac twice a day, for 30 seconds each time. Unfortunately, no statistically significant differences were noted between the group receiving placebo and the group administered the drug [38].

### 2.3. EGFR Inhibitors

Epidermal growth factor receptor (EGFR) is a protein belonging to the family of receptors of ErbB, which is present on the cell surface. EGFR is composed of an extracellular domain, which binds with the receptor ligand, transmembrane domain, and intracellular domain having tyrosine kinase activity. The ligands for the receptor are proteins from the growth factor family, which have a domain of structure homological to the epidermal growth factor (EGF). After connecting the ligand to receptor, a cell activation cascade is launched, which may contribute to transformation into a cell with potentially malicious phenotype, in case of excessive activity of the receptor. EGRF overexpression was confirmed in numerous types of neoplasms, also in more than 80% of HNSCC. This resulted in searching for agents that block the activity of EGFR, which would have a two-way action: creation of antibodies that block the receptor and development of low molecular weight inhibitors of tyrosine kinase (TKIs) [39, 40]. The former group comprises the following drugs: cetuximab, panitumumab, and zalutumumab; the latter: erlotinib and vandetanib.

The cetuximab monoclonal antibody found application for adjunctive treatment during radiotherapy in advanced forms of head and neck squamous cell carcinomas improving 5-year survival [41, 42]. In a study of Bonner et al. 5-year overall survival was 45.6% among patients receiving radiotherapy and cetuximab, whereas only 36.4% in the group treated with radiotherapy alone. The results of adding cetuximab to chemotherapy consisting of cisplatin or carboplatin combined with 5-fluorouracil are not so good. Cetuximab (Erbitux) also significantly increased the medium overall survival and the medium progression-free period. However, the median overall survival was still short, 10.1 months in the cetuximab group compared to 7.4 months in patients receiving chemotherapy alone [43]. These results come from the randomized phase III study EXTREME (the Erbitux in First-Line Treatment of Recurrent or Metastatic Head and Neck Cancer) and indicate that statistical significance of differences of overall survival observed in this study may not always mean clinical significance as statistically better overall survival was still clinically poor. Administered in monotherapy (for patients with cancer resistant to chemotherapy), cetuximab provided response in 13% of treated individuals [44]. A somewhat lower efficacy was demonstrated by the following two antibodies: panitumumab and zalutumumab. Panitumumab was administered to a large group of patients, in combination with chemotherapy (SPECTRUM study), while zalutumumab was tested on the group of patients with neoplasms resistant to chemotherapy [45, 46]. In both cases, prolonged progression-free survival (PFS) was achieved. Those results provided the basis for applying the EGR receptor inhibitors in studies concerning chemoprevention in HNSCC. In the studies performed on oral cavity carcinogenesis model, induced in mice by 4NQO (4-Nitroquinoline-1-oxide) carcinogen dissolved in water, a substantial reduction (69%) was demonstrated, of both dysplasia and squamous cell carcinoma by means of erlotynib, a low molecular weight inhibitor of tyrosine kinase domain of EGF receptor [47]. Unfortunately, such good results did not get repeated in the study performed on cetuximab application in humans, the EPOC study (Erlotinib Prevention of Oral Cancer). The patients that qualified for participating in the study belonged to a high risk group, as regards development of squamous cell carcinoma, due to advanced dysplasia, confirmed loss of heterozygosity (LOH), a potential risk marker of premalignancies turning malignant, in one of typical locations for head and neck cancerogenesis, or due to the history of head and neck carcinoma. A group of 150 patients with confirmed loss of heterozygosity qualified for the study, they were administered erlotynib in the doses of 150 mg/day, for 12 months, and were followed-up for another two years. The disease free survival (DFS) did not change, what is more, in about half of the subjects the erlotinib doses were reduced, due to adverse effects [48]. Much hope arises from the results of the study concerning vandetanib, conducted on animal model of carcinogenesis (4-NQO). Vandetanib is an inhibitor of two receptors: for Vascular Endothelial Growth Factor (VEGF), and for Epithelial Growth Factor (EGF). The application of vandetanib for 24 weeks in mice was linked with significant reduction of the percentage of dysplasia or cancer occurrence (from 96% in the placebo group to 28% in the group receiving the drug) [49]. Phase I studies were conducted, concerning the application of erlotinib as a chemopreventive agent in 12 patients diagnosed with advanced premalignancies, mainly leukoplakia, dysplasia of low, medium, and high degree, as well as cancer in situ. The therapy lasted for about 5 months and comprised combined oral application of erlotinib, in the doses 50, 75, and 100 mg as escalating dose, and oral administration of celecoxib, in the dose of 400 mg, 2 times a day for 6 months. The control was performed by means of biopsy after 3, 6, and 12 months. In 43% of patients the histological picture of lesions improved, while in 29% no further deterioration was noted. Also, reduced activity of EGF and p-ERK receptor was observed, which is one of the proteins of the MAPK/ERK signaling pathways, launched as a result of EGFR stimulation. Those results are a promising prognostic sign for further studies. The limitations of erlotinib doses were related to rash being an adverse effect [50].

### 2.4. PPARs

Peroxisome Proliferator-activated Receptors (PPARs) constitute a group of steroid nuclear receptors that may perform the role transcriptive agents, which regulate the expression of genes connected with the metabolism of carbohydrates, fats, and proteins, as well as with proliferation of cells and the course of inflammatory conditions. PPAR-*γ* (one of the kinds of PPARs) may act as suppressor of tumour growth. After activation, it binds with retinoid X receptor, which has inhibiting influence upon angiogenesis and proliferation, and stimulates cell differentiation. The compounds which are agonists of PPARs are also used in the treatment of diabetes, and have already completed the phase of preclinical studies in chemoprevention of head and neck carcinoma, with positive results. Pioglitazone and troglitazone have been studied in the mouse model of carcinogenesis (4NQO) and have proven to inhibit oral cavity carcinogenesis [51, 52]. Also, reduced expression of COX-2 in the studied tissues has been observed.

### 2.5. mTOR Inhibitors

Another group that may potentially have antineoplastic action comprises inhibitors of PI3K/Akt/mTOR signal transduction pathway. This intracellular pathway, important for the regulation of cell cycle, plays a role in the regulation of processes connected with survival and proliferation of cells. Excessive activity of its proteins is connected with amplification or mutation of genes encoding the serine/threonine protein kinase Akt, phosphoinositide 3-kinase (PI3K), as well as deletion or mutation of the phosphatase and tensin (PTEN) gene. Excessive expression of Akt may exert influence upon dysregulation of angiogenesis, inhibition of apoptosis, and promotion of migration. Inhibitors of serine/threonine protein kinase mTOR (mammalian target of rapamycin kinase); rapamycin (sirolimus) and its derivatives (temsirolimus and everolimus) are compounds from the group of macrolide antibiotics. Those compounds have antiproliferative and antiangiogenic action. They inhibit the growth and proliferation of tumour cells, as well as endothelium cells, fibroblasts, and smooth muscle cells of blood vessels. Their action has been confirmed in several studies, conducted on various animal models of HNSCC. In mice, rapamycin reduced lymphangiogenesis in primary tumours, as well as prevented the dissemination of neoplastic process to lymph nodes of the neck [53]. In another experimental model of HNSCC in genetically modified mice, rapamycin inhibited the transformation of premalignancies into malignant neoplasms, prolonging the survival of animals studied [54].

### 2.6. Vitamin E

Vitamin E is a group of chemical compounds soluble in organic fats, which includes tocopherols and tocotrienols. Tocotrienols are unoxidized forms of vitamin E, which demonstrate antioxidative activity. Vitamin E not only protects cells from oxidants, but also takes part in provision of nutrients to cells, strengthens blood vessel walls, and protects red blood cells from premature decomposition. For humans, the most vital role is that performed by *α*-tocopherol, the most biologically active form of vitamin E which, similar to retinoids, was the subject of many studies concerning chemoprevention. A randomized study conducted by Bairati et al., focused on the influence of supplementation with antioxidants soluble in fats, *α*-tocopherol and *β*-carotene, upon the occurrence of SPTs in patients with HNSCC stage I or II, undergoing radiotherapy. The plan was to administer both antioxidants during radiotherapy and for the following 3 years. During the study, administration of *β*-carotene was discontinued, whereas the supplementation with *α*-tocopherol was maintained. This was caused by ethical reasons, resulting from the report that emerged at that time, concerning increased incidence of lung cancer during supplementation with *β*-carotene. In patients receiving large doses of *α*-tocopherol (400 IU/d) increased mortality was observed, with reference to the group which did not get the supplementation [55, 56]. When mentioning that study, one should keep in mind the fact that one of the mechanisms behind the therapeutic effects of radiotherapy in the treatment of neoplasms is the generation of reactive forms of oxygen, via ionizing radiation, which damage tumour cells. Thus, the negative effects of vitamin E application may be caused by combining its supplementation with radiotherapy.

### 2.7. Folic Acid

Reduction of the amount of folic acid influences the lack of balance in the synthesis of uracil and thymine, pyrimidine bases that form RNA and DNA, which—in turn—causes disturbances in DNA synthesis and its repair. What is more, deficiency of folic acid contributes to problems with DNA methylation and difficulties with control and expression of proto oncogenes.

Folic acid deficiency is connected with increased exposure to neoplasm occurrence. A research was conducted on 745 patients with cancer of the oral cavity and pharynx, who responded to a questionnaire concerning eating habits, with particular attention paid to the consumption of food containing folic acid. The results obtained were compared with results from the control group of 1772 subjects who were hospitalized due to other ailments, not connected with neoplasia. It was concluded that the consumption of folic acid in diet, in medium or big amounts, may prevent the occurrence of neoplastic lesions in the mouth cavity, even in case of alcohol abuse, alcohol being an agent which fosters the formation of neoplasms [57].

### 2.8. Natural Agents

#### 2.8.1. Curcumin

One of the most thoroughly studied substances of natural origin is curcumin. Curcumin (or turmeric, Latin: C*urcuma longa*) is a plant of the ginger family, commonly used as a condiment. The chemically active substance included in it is the light yellow flavonoid-curcumin (diferuloylmethane). Its pleiotropic activity in chemoprevention comprises regulation of several intracellular signal transduction pathways at various levels, such as transcription factors: NF-*κ*B, (nuclear factor *κ*-light-chain-enhancer of activated B cells), Akt, MAPK, Nrf2, Notch-1, JAK/STAT. It also influences the expression of many important proteins: p53, *β*-catenin, EGFR, adhesion molecules, AP-1 (activator protein-1) protein complex, and cyclin D1. Curcumin also causes increased expression of proteins of the caspase family, as well as reduced expression of antiapoptotic genes Bcl-2 and Bcl-X(L) [58, 59]. Curcumin enhances apoptosis via mitochondrial pathway, too [60, 61]. It also reduces the expression of COX-2. In an experimental study curcumin proved to be the most effective flavonoid protecting otopharyngeal mucosa against a damage induced by tobacco smoke [62]

In preclinical studies performed on rats, curcumin was applied for chemoprevention of neoplastic activity of 4-NQO, it was administered in drinking water for 8 to 12 weeks, in the doses of 50 ppm. In the groups in which rats received curcumin in the amount of 100 mg/kg of body mass, for 12 weeks, a significant reduction of PCNA, Bcl2, SOCS-1 e-3 and STAT3 expression was noted, which are characteristic biomarkers for oral cavity neoplasms. What is more, curcumin reduced the number of atypical cells in microscopic examinations, and reduced the expression of genes related to EMT (Epitelial to Mesenchymal Transition), a process which is key for cancer cells to obtain the metastasis ability [63]. Several clinical studies with the use of curcumin have been conducted. In one of them, patients with leukoplakia in the mouth cavity received increasing doses of curcumin extract, starting with 500 mg/day and reaching the highest tolerated dose of 8,000 mg/day, the administration period being 3 months. No toxicity was noted for the highest dose. After the treatment, biopsy of the lesion was performed, which demonstrated that only in one of seven patients did the lesion turn malignant [64]. Similar results have been obtained in a larger study, comprising 25 patients for each disease: leukoplakia, lichen planus, or submucous fibrosis. Reduction of pain was achieved, as well as gradual reduction of the lesion size, up to complete healing in a large percentage of cases. The subjects also had the vitamin C and E level in serum and saliva measured, which was elevated after the treatment, which indicates the antioxidative action of curcumin extract [65]. Unfortunately, the factor that limits the application of curcumin in NHSCC chemoprevention is its relatively low bioavailability, in comparison to other tissues, e.g., those of the stomach and intestines. In the study conducted for cell lines CCL23, CAL27, and UM-SCC1 of head and neck carcinoma it was demonstrated the curcumin inhibited, in a dose-dependent manner, the growth of all three cell lines, and reduced the expression of NF-*κ*B and cycline D1. In the other part of the study the authors demonstrated that curcumin in the form of ointment inhibited the growth of tumours in mice, who had neoplastic cells of the CAL27 line grafted [66].

#### 2.8.2. Green Tea Extract

Green tea (*Camellia sinensis*) contains 4 main polyphenols, also referred to as catechins: 10-15% (-)-epigallocatechin gallate (EGCG), 6-10% (-)-epigallocatechin (EGC), 2-3% (-)-epicatechin gallate (ECG), and 2% (-)-epicatechin (EC). The first three have antineoplastic properties, whereas EC is not active. In a 9-year prospective study conducted in Japan, comprising a group of 8522 persons over 40 years of age, it was noted that drinking more than 1200 ml (10 Japanese tea cups) of green tea a day reduces the relative risk of cancer. That effect was noticed in both women and men. However, taking into account in statistical analysis the risk factors of HNSCC, such as consumption of alcohol and smoking cigarettes, resulted in the fact that the protective effect of green tea in men has not reached the level of statistical significance [67, 68].

EGCG is the most thoroughly studied ingredient of green tea. This compound performs its chemopreventive action not only via antioxidant properties, but also influences various signaling pathways in cells. It inhibits the activation of tyrosine kinases of membrane receptors: IGFR-1 (insulin-like growth factor receptor 1), HER1 (human epidermal growth factor receptor 1, also called EGFR (epidermal growth factor receptor)) and HER2 (human epidermal growth factor receptor 2), as well as VEGFR (vascular endothelial growth factor receptor). It inhibits the following kinases: Akt, ERK1/2 (extracellular signaling-regulated kinase 1/2), MAPK (mitogen-activated protein kinase). It also influences the activity of phosphoinositide-3 kinase (PI3K) and mTOR (mammalian target of rapamycin kinase). EGCG also modulates the activity of NF*κ*B transcriptive factor. Those activities result, among others, in reduced COX-2 expression, activation of protein p53, and induction of apoptosis. EGCG reduces the generation of metalloproteinase MMP-2 and -9 in cancer cells, which influences reduction of tumour invasiveness [69–72].

Pisters et al. conducted phase I research using green tea extract (GTE) for patients with neoplasms, including 19 people with HNSCC. The maximum tolerance dose of GTE was 4.2 g/m^2^ of body surface, once daily, or 1.0 g (the equivalent of 7-8 Japanese cups) 3 times a day, for 12 weeks. That research reported no significant clinical response [73]. In randomized study phase II the high risk patients with oral premalignant lesions (OPLs) received GTE for 12 weeks, in doses of 500, 750, or 1000 mg/m^2^ of body surface. For all three doses of GTE, a higher percentage of clinical responses was noted than in placebo group, however, in no study group is that difference at the level of statistical significance. In the joint analysis of 3 doses applied, clinical response was elicited in 50% of patients, still—in comparison with placebo group—that difference was not statistically significant [74]. In the subsequent study devoted to EGCG, Fujiki demonstrated that this compound inhibits the expression of genes encoding markers of cancer stem cells (CSCs). EGCG also inhibited the ETM process in human CSCs. That research indicates further mechanisms of antineoplastic activity of EGCG [75].

#### 2.8.3. Genistein

Genistein (4,5,7-trihydroxyisoflavone) is a natural isoflavonoid from soya beans. The research with the use of HN4 cell lines of head and neck carcinoma demonstrated that pleiotropic activity of genistein causes induction of apoptosis and inhibition of cancer cell growth, by stopping the cell cycle in the G_2_/M phase. Genistein also proved to inhibit the invasiveness of tumour cells. This effect was due to the inhibition of expression of c-erbB-2 protein and MMP-2 and -9 metalloproteinases. Genistein also induced telomere shortening in the examined line of neoplastic cells. Also the ability of binding transcriptive factor NF*κ*B with DNA diminished significantly [76, 77]. In his previous study, Alhasan demonstrated that genistein action is specific for neoplastic cells, and does not take place in regular keratinocytes [78]. The research, conducted on SCC-25 cell line of oral cavity squamous cell carcinoma demonstrated that genistein may—also in that cancer—limit cell divisions by arresting the cell cycle in G_2_/M phase. On the other hand, as opposed to the effects noted in research with the use of HN4 cell line, in the culture of cell line SCC-25 genistein did not demonstrate proapoptotic action. What is more, genistein activity was connected with inhibition of COX-2 activity, which influences the reduction of PGE_2_ prostaglandin production in a similar way to what celecoxib does, while its antineoplastic activity was less significant than that of celecoxib or indomethacin (nonspecific inhibitor of COX) [79].

#### 2.8.4. Bowman-Birk Inhibitor

During carcinogenesis, imbalance occurs between the activity of proteases and their inhibitors, where proteolysis prevails. This discovery provided impetus to search for new targets for chemoprevention of neoplasms and attempts of applying inhibitors of proteases.

Bowman-Birk inhibitor (BBI) is an inhibitor of serine proteases. It was discovered in 1940 by Bowman in soya beans. It has the ability to inhibit chymotrypsin and trypsin. It is assumed that BBI also possesses antioxidant properties and influences DNA repair, as well as the metabolism of arachidonic acid [80]. Armstrong conducted clinical studies phase IIa and IIb with BBI concentrates, on patients with oral cavity leukoplakia. In phase IIa study, conducted on 32 patients, the researcher observed reduction of leukoplakia surface after 1 month of treatment. In phase IIb study, comprising 132 patients and concerning longer application of BBI concentrate, lasting 6 months, a regression of lesion surface by 20.6%, was observed, yet the difference, in comparison with placebo (regression by 17.1%), was not statistically significant. That absence of significance of differences was a result of application of placebo which is of plant origin and—not demonstrating proteolytic activity—could contain substances with other chemopreventive action. Also the BBI concentrate storage conditions and storage time could have influenced the reduction of its activity [80, 81].

#### 2.8.5. Sulphoraphane

The research is in progress as regards the activity of sulphoraphane (SFN) that is a substance which is present mainly in broccoli and other cruciferous vegetables. Those vegetables contain compounds called glucosinolates which, under the influence of myrosinase enzyme, are transformed into isothiocyanates. That process takes place after destruction of the plant cell, it is also catalyzed by intestinal bacteria. SFN is the most important in that group of compounds. It is formed from glucoraphanin precursor (a stable *β*-thioglucose). The highest concentration of glucoraphanin may be found in broccoli seeds and sprouts. The proposed mechanism of sulphoraphane action is the regulation of NRF2 (nuclear erythroid 2-related factor), which is the main protein protecting cells from oxidative stress. SFN also demonstrates anti-inflammatory and proapoptotic action, it influences histone proteins as well. Another important mechanism of SFN activity is the inhibition of cytochrome CYP2E4, which oxidizes benzo[a]pyrene and other polycyclic aromatic hydrocarbons (PAH) to epoxydiols that are better soluble in water, which may cause mutations and initiate carcinogenesis [59, 82, 83].

#### 2.8.6. Resveratrol

Resveratrol (3,5,40-trihydroxy-trans-stilbene) belongs to natural polyphenols and is present in many plants, e.g., in raspberries, strawberries, currants, huckleberries, peanuts and grape skin, particularly red grape skin. The influence of resveratrol upon precancerous condition and oral submucous fibrosis was investigated.

The pathogenesis of submucous fibrosis involves excessive expression of ZEB1 (zinc finger E-box binding homeobox 1). Resveratrol inhibits the activity of ZEB1 and may help in the treatment of that ailment or prevent the lesion from becoming malignant [84]. Its antineoplastic properties have been investigated in several preclinical studies on cell lines. Resveratrol inhibited the growth and proliferation of cells from HNSCC cell lines [62].

#### 2.8.7. Preparation from Lyophilized Strawberries

Strawberries (*Fragaria* ×* ananassa*) contain several components with potential chemopreventive activity, including vitamins A, C, and E; folic acid, calcium, selenium, *β*-sitosterol, ellagic and ferulic acids; flavonols such as kaempferol and quercetin; and multiple anthocyanins. Research concerning the influence of lyophilized strawberries upon the prevention of development of neoplastic lesions in the oral cavity has been conducted on hamsters. Cancerogenesis was induced by swabbing malar bags with 0.2% DMBA (7,12-dimethylbenzanthracene) for 6 weeks. Simultaneously, the hamsters received in their diet the 5% or 10% solution of lyophilized strawberries (LS), before, during, and after neoplasia induction. After completing the 12-week study cycle, significant reduction of the number of formed neoplastic lesions was found, as well as reduction in their progression level, along with reduction of incidents of severe or medium level dysplasia. Moreover, molecular studies confirmed the influence of LS upon the expression of genes responsible for the development of oral carcinomas [85].

#### 2.8.8. Black Raspberries

Black raspberries (*Rubus occidentalis*; BRB) are rich in many bioactive components, anthocyanins, and additional bioactive plant polyphenols including: ellagic acid, ferulic acid, coumaric acid, quercetin, and phytosterols; as well as other micronutrients including: folic acid, selenium, and vitamin C, which may act individually or in combination to inhibit cancer development in various ways. Black raspberry extract demonstrated beneficial chemopreventive activity in animal models of HNSCC. Both in golden hamsters, in which neoplastic lesions were induced by means of DMBA [86, 87], and in rats subjected to 4NQO activity [88], the diet enriched with BRB extract resulted in beneficial results, reducing the number of animals that developed cancer, the size of the tumour and its multiplicity.

Knobloch et al. conducted clinical studies of phase 0 on 38 patients for whom biopsy confirmed the oral squamous cell carcinoma (OSCC). For the time remaining before the planned surgery (14 days on the average) the patients received tablets containing lyophilized powder from black raspberries. The excised cancer tissue of the patients receiving BRB revealed significant reduction of expression of genes encoding proteins, important for the survival of cancer cells (AURKA, EGFR, or BIRC5), as well as proinflammatory agents NFKB1, PTGS2, whose expression in OSCC cells is substantially exceeded. What is more, the presence of BRB active substances such as cyanidin-3-rutinoside and cyanidin-3-xylosylrutinoside was detected in OSCC cancer tissue, which is the evidence of their accumulation in the target tissue [89]. Topical application of a mucoadhesive freeze-dried BRB gel was tested in a placebo-controlled, multicenter study comprising 40 patients with oral premalignant intraepithelial lesions. Topical application of BRB significantly reduced lesions size, histologic grade and LOH events, whereas in placebo group a progression of lesions size was observed [90].

Both strawberries and black raspberries contain ellagic acid (EA), which is considered to be one of the substances responsible for the beneficial chemopreventive activity of both extracts. In the study reported, conducted by Oghumu et al. on rats, a comparison was made between the result of adding EA only to fodder and adding BRB extract. EA alone inhibited the development of neoplasia, but not as strong as in case of BRB extract [88].

### 2.9. Biochemoprevention

Interferon-*α in vitro* inhibits the proliferation of cells and their differentiation. Combined application of interferon-*α*, retinoic acid, and *α*-tocopherol is referred to as biochemoprevention. In prospective phase II studies, as well as studies planned as tertiary prevention (SPTs prevention) the joint application of 13-cis-retinoic acid (isotretinoin), in the doses of 50 [91] or 100 mg/m^2^/day [20] and *α*-tocopherol in the dose of 1200 IU/day, as well as interferon-*α* in the doses of 3 × 1 million IU/m^2^ for one year [91] or 2 years [20, 92] gave promising results, both in patients with premalignant lesions [20], and with HNSCC [91, 92]. The use of biochemoprevention in patients after surgical excision and/or radiotherapy of HNSCC stage III or IV resulted in five-year survival of 81.3% of the patients [92]. In the study assessing the influence of biochemoprevention upon premalignant lesion, full remission was attained in 47% of patients after 6 months of treatment, and in 50% after a year of treatment, in case of lesions located in the larynx area. On the other hand, in case of lesions located in the mouth cavity the results were much poorer, with mere 9% of remissions after 6 months [20].

### 2.10. Adenovirus ONYX-15

Yet another interesting attempt at chemoprevention of HNSCC was the development of modified adenovirus ONYX-15, which replicates selectively only in cells with mutated gene p53, destroying them. As mutations of gene p53 occur in some 40-50% cases of HNSCC and in some 45% of dysplastic lesions, the idea was born to apply adenovirus ONYX-15 in patients with dysplasia of the oral cavity. Resolution of dysplasia was observed in 7 of 19 patients who completed the study. Despite the fact that this kind of treatment was well tolerated, the achieved percentage of positive response to treatment, and the transient nature of the improvement demand much caution in assaying the method [93, 94].

## 3. Assessment of Chemoprevention Efficiency

Despite almost 40 years of research in chemoprevention of HNSCC, no standards have been developed for that method as yet. That is probably due to several reasons, as a few facts should be considered in case of chemoprevention of HNSCC. The notion of HNSCC comprises squamous cell carcinomas with different locations: larynx, mouth cavity, nasal cavity, lips, nasal/paranasal sinuses, the tongue, salivary glands. Although in the classification of cancers they make up one group, the results of treatment and chemoprevention of those tumours and premaligancies are different for specific anatomic locations. The confirmation of that postulate can be found in the results from the study of Papadimitrokopolous [20], referred to above, in which the results of biochemoprevention were distinctly different for lesions located in the larynx and in mouth cavity.

Another factor influencing the results of studies concerning chemoprevention is its type. It would be difficult to indiscriminately compare the effects of primary prevention—in which several factors should be taken into account, related both to protection and to risk—with the effects of secondary of tertiary prevention. It should be accounted for that the cell biology in a premalignant lesion may differ significantly from a tumour cell. The term OPMLs comprises lesions with various degrees of dysplasia. Intraepithelial neoplasia (IEN) is pointed out ever more. As lesions of such type macroscopically resemble healthy tissue and are separated from OPMLs, American Association for Cancer Research Task Force on Cancer Prevention introduced the term “molecular IEN”. This population of cells may be responsible for SPTs formation. At present, much attention is paid to the need for more detailed identification of high risk patients, for whom greater advantages from chemoprevention may be expected. The very finding of OPML in a patient begins to be insufficient. Finding suitable molecular markers is needed, which shall allow qualifying patients for appropriate chemoprevention. For more detailed discussion of those issues refer to literature items [12, 95].

Analyzing the results of various studies concerning chemoprevention of HNSCC, one should take into account yet another factor, that is time. In different studies, chemoprevention has been applied for different periods: 3, 6, 12 months, 2, 3, 5, or even 7 years [11, 29, 30, 37, 81, 90, 96, 97]. In case of such big differences in the time of applying chemoprevention, it is hard to compare results. Time also matters in studies, if we take into account the length of follow-up during which the effects of chemoprevention are assessed, both regarding prevention of OPMLs in HNSCC, and prevention of SPTs. The follow-up period may be measured in various ways, either including the time during which chemoprevention was applied, or after it had been completed. One should also keep in mind, which was pointed out by some researchers, the phenomenon of transient clinical response (e.g., leukoplakia regression). The assessment of durability of positive results of chemoprevention is possible in case of sufficiently long follow-up period.

Messadi [11] indicates another aspect which should be noted when analyzing results of individual studies concerning chemoprevention of HNSCC, namely the age of subjects. The period in which the risk of malignant transformation occurs, thus the period in which chemoprevention should be applied and patients should be monitored for early detection of HNSCC, is different for middle-aged and elderly patients. This is yet another factor that should be considered when developing the guidelines concerning the application of chemoprevention. Messadi also suggests those guidelines to be differentiated for individual countries, taking into account such cultural factors as diet, as well as consumption of alcohol and tobacco.

In discussing the efficacy of agents used in chemoprevention of HNSCC, one should take into account their pharmacokinetic properties, e.g., solubility, which influence their bioavailability. This is taken into account in preparation of pharmaceuticals, for example drugs administered by mouth, as well as applied topically [98]. In case of prevention of oral carcinoma, one should take into account the anatomical and physiological specificity of that location. The agent which will be applied topically, e.g., for swabbing OMPLs or rinsing the mouth, is quickly washed out by saliva, and tongue movements speed that process even more. A strong challenge is the problem of how to prolong the contact time of a substance acting on a surface with OPMLs. One of the attempts to solve the problem was a suggestion of using a gauze soaked with the appropriate medicine.

Another direction of assuring a better bioavailability of the medicine applied in chemoprevention is the application of nanotechnology, developing ever quicker. Via suitable formation of the nanoparticles as drug carriers, one can control their penetration through cell membranes, enabling a targeted delivery of medicines to pathologically changed cells. This technology also allows to regulate the drug release rate. Both directions of action may result in reducing the adverse effects. An example of such a strategy is the development of a truncated, octahedral nanocarbon, sized 4-5 nm. A conjugate of that nanoparticle with polyethyleneimine-poly(ethylene glycol) may serve as a nanocarrier of a COX-2 inhibitor, celecoxib [99]. A form of nanocarrier was also developed for isotretinoin, as a lipid nanocarrier having the diameter of about 70-80 nm. Isotretinoin in such a shape not only reduced its sensitivity to light, but also—after topical application—reduced the penetration of the medicine to the systemic circulation, as well as reduced the adverse effects [100]. Jayaprakasha invented nanoencapsulation of curcumin with whey protein. In experimental studies on colon cancer and prostate cancer cell lines this formula gave better results than plain curcumin [101]. Promising results were also obtained in a study of a novel polymeric nanocarrier-curcumin [102, 103]. Siddiqui introduced the notion of “nanochemoprevention”, which describes the new direction of studies concerning drugs used in chemoprevention. Nanoformulation of resveratrol and EGCG has already been developed [104–106]. For curcumin, yet another manner of application has been developed, namely polymeric implants that release the drug gradually. Trials on animals demonstrated that this way of drug application results in better bioavailability than oral administration [107, 108].

## 4. Safety Issues concerning Chemoprevention of HNSCC

At first sight, it may seem that chemoprevention of cancer is a method in which compounds of natural origin are mainly used, so it is safe. One should keep in mind, however, that among the substances applied in chemoprevention there are compounds with strong biological activity. Utmost care should applied when using retinoids. This is not a homogeneous group of compounds (Table 2). Besides chemoprevention of HNSCC, retinoids are also applied for therapeutic purposes in dermatology, hematology, and oncology. Adverse effects accompanying the application of retinoids may affect some various fields of medicine.

### 4.1. Ophthalmic Adverse Effects of Applying Retinoids

When applying retinoids in various fields of medicine, attention is most frequently paid to the side effects concerning the organ of vision. Those side effects differ, depending on the retinoid applied. Isotretinoin is known to affect the Meibomian glands. A regular lacrimal film is composed of three layers: (i) the internal one, aqueous layer, produced by lacrimal glands, (ii) the middle one, mucous layer, produced by goblet cells of the conjunctiva, and (iii) the external one, lipid layer,* meibum*, produced by Meibomian glands. The lipid layer provides a smooth optical surface, delays the evaporation of tears, provides a barrier protecting from impurities from the skin in the eye area, maintains the stability of tear film, as well as structural and refractive coherence of the eye surface. A vital component of the lipid layer is the oily secretion from the Meibom's glands. Isotretinoin inhibits the differentiation of cells producing the oily secretion, at the same time it stimulates the proliferation of epithelium lining the ducts leading from Meibom's glands, narrowing their lumen. As a result of those activities, isotretinoin causes reduced secretion of the oily secretion (*meibum*). Isotretinoin leads to atrophy of Meibonian glands. Although the amount of tears produced is not reduced, the deficiency of lipids leads to disturbances in the lacrimal film, symptoms of dry eye syndrome, clinical manifestations of inflammation of eyelid margins, as well as disturbances of the eye surface. The most frequent side effects after the treatment with retinoids are: dry eye syndrome, as well as inflammation of eyelid margins (*blepharitis),* and conjunctivitis* (blepharoconjunctivitis)*, which in 20-50% develop over the period of 3-5 weeks after the onset of treatment [109–111].

Some researchers presume that the disorders of lacrimal film functions may be caused by the presence of isotretinoin and its metabolites in tears, and direct disturbance of lacrimal film stability [109, 110]. It should be remembered that isotretinoin applied topically is absorbed and gets to the systemic circulation. That way of isotretinoin administration does not protect completely from adverse effects.

Fenretinide originated in the USA in the late 1960s. Although it is considered less toxic than other analogs of retinoic acid, it still has certain ophthalmic adverse effects. It interferes with the binding of retinol with retinol binding protein (RBP). Retinol is a hydrophobic and lipophilic compound. In plasma, it can be transported after binding with RBP. A disturbance of that process results in reducing the retinol level in blood plasma and its bioavailability in the retina. A consequence of such activity of fenretinide is the disturbed adaptation to seeing in the dark, which may have a manifested form, or—even in 50% of the cases— an asymptomatic form, detectable only in dark adaptometry examination using the Goldmann-Weekers adaptometer [112]. The level of disturbances is inversely proportional to the level of retinol in serum. The problems with adaptation to seeing in the darkness, or even nyctalopia, occurring when fenretinide is administered for various indications, are reversible as a rule, after fenretinide administration is discontinued and vitamin A supplementation is started [112, 113]. Marmor points out that large doses of fenretinide may completely block the rod cell functions, yet that is reversible after discontinuation of treatment. Fenretinide seems not to influence the functioning of cone cells [114]. Most of the side effects of treatment are reversible, yet in some 4.4% of patients those symptoms are maintained after discontinuation of treatment [115]. Also, a case of multilayered retinal hemorrhage (large sub- and intraretinal hemorrhage) has been described, which occurred in a woman patient treated with fenretinide administered intravenously due to hairy-cell leukemia [115].

### 4.2. Other Adverse Effects of Retinoid Administration

The teratogenic action of retinoids is the most serious adverse effect of their administration. For that reason, oral administration of retinoids is strictly contraindicated in pregnant or lactating women. When such agents are administered to women in reproductive age, efficient contraception should be applied during treatment and after its completion. In case of oral acitretin that period may be even up to two years long [116]. Other adverse effects of retinoid application include: excessive dryness of nasal mucous membrane and lips (xeromycteria and xerochilia), xerodermia, and baldness. Cases of depression have also been described in people staying on isotretinoin.

### 4.3. Adverse Effects of Application of Other Agents Used in Chemoprevention of HNSCC

When interferon-*α* is applied, one can expect the occurrence of such side effects as mild to moderate headaches, ostealgia, and myalgia (influenza manifestations), hypertriglyceridemia, and mild hematological disturbances [91]. When selective inhibitors of COX-2 are applied, one should be aware of the risk of embolic-thrombotic disturbances, e.g., cerebral stroke. Vertigo has also been reported as a consequence of their administration [11, 37]. Thiazolidinedione, in turn, may increase the risk of circulatory insufficiency. In the studies conducted by Tsao et al., the application of green tea extract resulted in reported insomnia and nervousness [11, 74]. Erlotinib caused dermatological problems, diarrhea, fatigue, and mucositis [48]. Reports from studies concerning other agents used in chemoprevention of HNSCC do not mention significant adverse effects. However, various phytochemicals supposed to have chemopreventive properties against others cancers may have toxic effects including even carcinogenicity [117].

## 5. Conclusion

At present, no uniform standards or guidelines exist as concerns chemoprevention of HNSCC. Studies conducted on cell cultures of neoplastic cell lines of HNSCC and on animals give promising results, yet they do not find full confirmation in clinical studies on humans. The increasingly better understanding of the molecular mechanism of cancerogenesis allows to identify new targets, in which this process can be stopped or reversed by means of a suitable chemopreventive agent. Also the mechanisms of substances studied have been ever better recognized. Despite that, there are still not many substances with potentially anticancerogenic action that have been subject to phase 3 clinical studies and the few substances that have undergone such studies turned to be less effective than forecasted.

The better our understanding of carcinogenesis mechanisms and premalignancy lesions biology, the more urgent the finding of suitable clinical, histological, molecular, or genetic markers, which will allow for individually targeted chemoprevention, thus enhancing its efficacy. Also, further studies are needed to find markers, which will allow taking decisions concerning termination or further continuation of chemoprevention [11].

An important role in the etiopathogenesis of head and neck carcinoma is played by infection with human papilloma virus (HPV). Chemoprevention of HPV-positive head and neck cancer is a relatively less profoundly studied subject. Much indicates that such carcinomas do not respond to the compounds studied so far [118]. The increasing percentage of HPV-positive HNSCC calls for the development of specific chemoprevention strategies for such a type of carcinoma.

The attention of scientists is focused, to a large extent, upon compounds of natural origin, as they possess many features of an ideal chemopreventive agent, such as absence of adverse effects or but a slight presence of them, which allows for long term application, wide availability, and low cost/price. Research will prove whether they will also demonstrate sufficient efficacy. The development of new technologies for provision of the pharmaceutical forms of them, particularly the nanocarriers, creates new hopes for further improvement of chemoprevention of HNSCC.

## Figures and Tables

**Table 1:** Inhibitors of carcinogen induced neoplasia (taken from [18] and modified).

**Blocking agents**	**Suppressing agents**
Phenols	Retinoids
(i) Ellagic acid	Carotenoids
(ii) Caffeic acid	Selenium salts
(iii) Ferulic acid	Proteases inhibitors
(iv) *p*-hydroxycinnamic acid	Inhibitors of arachidonic acid metabolism
Indols	Cyanates and isocyanates
Aromatic isothiocyanates	Phenols
Coumarins	Plant sterols
(i) Coumarin	Methylated xanthines
(ii) Limettin	(i) Caffeine
Flavones	Others
Dithiothiones	(i) Dehydrepiadrosterone
Diterpens	(ii) Fumaric acid
Ditiocarbamates	
Phenothiazines	
Barbiturates	
Trimethylquinolines	

**Table 2:** Retinoids and compounds blocking their metabolism used in therapy or clinical studies.

	**Retinoids and compounds blocking their metabolism**
1.	Retinoids of natural origin (first generation of retinoids)
	(i) Retinol
	(ii) Retinaldehyde
	(iii) Retinoic acid
	(a) All-trans-retinoic acid (ATRA)
	(b) 9-cis retinoic acid
	(c) 13-cis retinoic acid (tretinoin, isotretinoin, alitretinoin)

2.	Synthetic monoaromatic retinoids (second generation of retinoids)
	(i) Acitretin
	(ii) Etretinate (withdrawn from the market)
	(iii) Motretinid

3.	Synthetic poliaromatic retinoids (third generation of retinoids)
	(i) Adapalene
	(ii) Tazaroten
	(iii) Bexarotene

4.	Atypic retinoids
	(i) Fenretinide (4-HPR; *N-*(4-hydroxyphenyl)retinamide)

5.	Retinoic acid metabolism blocking agents (RAMBAs)
	(i) Liarozole (inhibitor of cytochrome p450 acid 4-hydroxylase)
